# Characterization of a *Toxoplasma gondii* calcium calmodulin-dependent protein kinase homolog

**DOI:** 10.1186/s13071-016-1676-1

**Published:** 2016-07-21

**Authors:** Kentaro Kato, Tatsuki Sugi, Hitoshi Takemae, Ryo Takano, Haiyan Gong, Akiko Ishiwa, Taisuke Horimoto, Hiroomi Akashi

**Affiliations:** National Research Center for Protozoan Diseases, Obihiro University of Agriculture and Veterinary Medicine, Inada-cho, Obihiro, Hokkaido 080-8555 Japan; Department of Veterinary Microbiology, Graduate School of Agricultural and Life Sciences, The University of Tokyo, 1-1-1 Yayoi, Bunkyo-ku, Tokyo, 113-8657 Japan

**Keywords:** Calcium calmodulin-dependent protein kinase homolog, GAP45, Phosphorylation, *T. gondii* CaMK-related kinase, *Toxoplasma gondii*

## Abstract

**Background:**

*Toxoplasma gondii* is an obligate intracellular parasite of the phylum Apicomplexa and a major pathogen of animals and immunocompromised humans, in whom it causes encephalitis. Understanding the mechanism of tachyzoite invasion is important for the discovery of new drug targets and may serve as a model for the study of other apicomplexan parasites. We previously showed that *Plasmodium falciparum* expresses a homolog of human calcium calmodulin-dependent protein kinase (CaMK) that is important for host cell invasion. In this study, to identify novel targets for the treatment of *Toxoplasma gondii* infection (another apicomplexan parasite), we sought to identify a CaMK-like protein in the *T. gondii* genome and to characterize its role in the life-cycle of this parasite.

**Methods:**

An in vitro kinase assay was performed to assess the phosphorylation activities of a novel CaMK-like protein in *T. gondii* by using purified proteins with various concentrations of calcium, calmodulin antagonists, or *T. gondii* glideosome proteins. Indirect immunofluorescence microscopy was performed to detect the localization of this protein kinase by using the antibodies against this protein and organellar maker proteins of *T. gondii*.

**Results:**

We identified a novel CaMK homolog in *T. gondii*, *T. gondii* CaMK-related kinase (TgCaMKrk), which exhibits calmodulin-independent autophosphorylation and substrate phosphorylation activity. However, calmodulin antagonists had no effect on its kinase activity. In *T. gondii*-infected cells, TgCaMKrk localized to the apical ends of extracellular and intracellular tachyzoites. TgCaMKrk phosphorylated TgGAP45 for phosphorylation in vitro.

**Conclusions:**

Our data improve our understanding of *T. gondii* motility and infection, the interaction between parasite protein kinases and glideosomes, and drug targets for protozoan diseases.

## Background

*Toxoplasma gondii* is an obligate intracellular parasite of the phylum Apicomplexa and a major pathogen of animals and immunocompromised humans, in whom it causes encephalitis [1, 2]. In humans, ingested *T. gondii* cysts release asexually reproducing bradyzoites that differentiate into tachyzoites, which propagate the infection by spreading through the body via the blood and lymphatic systems. While the immune system can normally clear a *T. gondii* infection, immunocompromised individuals, such as those infected with human immunodeficiency virus, have trouble doing so and can develop severe toxoplasmosis [3]. Although drugs to treat toxoplasmosis are available, they are poorly tolerated, have severe side effects, and are ineffective against chronic *Toxoplasma* infections [4, 5]. Therefore, new drugs are urgently needed. To discover new drug targets, we must first understand the mechanism of tachyzoite invasion. Such knowledge may also benefit the study of other apicomplexan parasites.

Our laboratory and others have demonstrated the importance of parasitic kinases for *T. gondii* [6–10]; however, no kinase function has been found to be of critical importance in the primary host, felines. For tachyzoite invasion of a host cell, many kinases are called to action, including *T. gondii* calcium-dependent protein kinase 1 (TgCDPK1) [7, 11–13], *T. gondii* cyclic GMP-dependent protein kinase [14], and TgCDPK1_2 [8]. TgCDPK1 also participates in the egress of tachyzoites from infected cells [12]. Additional *T. gondii* protein kinases are involved in host manipulation, cell cycle regulation, and functions required for growth, stress responses, and the transition from tachyzoite to bradyzoite [15]. Thus, given their level of involvement in many aspects of the parasitic life-cycle, the kinases encoded by the parasite genome are obvious potential drug targets.

The motility of *T. gondii* tachyzoites is activated by an increase in the cytosolic Ca^2+^ concentration [16], which occurs as the parasites egress from the host cells [17, 18]. This increase in Ca^2+^ concentration causes the parasites to secrete adhesion molecules from its microneme [19]. An actin/myosin-based motor complex, the glideosome [20], powers parasite motility and is a conserved feature of apicomplexans [21]. The glideosome of *T. gondii* is a macromolecular complex that includes myosin A, myosin light chain (TgMLC1), *T. gondii* glideosome-associated protein 50 (TgGAP50), TgGAP45, aldolase 1, and actin 1 (TgACT1) [20]. TgGAP40, TgGAP70, and TgGAP80 are also glideosome components [22, 23]. The glideosome, which is located between the parasite’s plasma membrane and its inner membrane complex, mediates motility, migration, host cell invasion, and egress. In *T. gondii* and another apicomplexan family member, *Plasmodium falciparum*, the phosphorylation of glideosome components within the tachyzoite, or merozoite in the case of *P. falciparum*, occurs during invasion and helps control gliding, invasion, and egress. In *T. gondii*, the phosphorylation of Ser^163^ and Ser^167^ in TgGAP45 marks the final step in glideosome assembly [24]. Structural modeling studies have shown that Ser^163^ and Ser^167^ are Ca^2+^-insensitive and that Ser^184/5^ and Thr^189^ are Ca^2+^-sensitive phosphorylation sites on TgGAP45 [25]. PfCDPK1 localizes to the periphery of merozoites where it acts on myosin A tail domain-interacting protein (MTIP) and PfGAP45 at the inner membrane complex. PfCDPK1 can also phosphorylate MTIP and PfGAP45 in vitro [26].

We previously characterized *P. falciparum* protein kinase 2 (PfPK2), which is a unique homolog of human Ca^2+^ calmodulin-dependent protein kinase (CaMK) [27]. PfPK2 phosphorylates its substrate in a Ca^2+^- and calmodulin-dependent manner. In the present study, we identified a homolog of PfPK2 in *T. gondii*, *T. gondii* CaMK-related kinase (TgCaMKrk) (ToxoDB ID: TGME49_315190; GenBank accession number: AB699221), which exhibits autophosphorylation and histone phosphorylation activity. However, calmodulin antagonists had no effect on its kinase activity. We further show that TgCaMKrk is expressed in *T. gondii*-infected cells and localizes to the apical ends of extracellular and intracellular tachyzoites, and that it specifically targets TgGAP45 for phosphorylation in vitro.

## Methods

### Target cells and parasites

Tachyzoites of *T. gondii* RH strain were used in this study. The parasite was maintained in monolayers of Vero cells cultured in Dulbecco’s modified Eagle’s medium (DMEM) containing 7.5 % fetal calf serum (FCS), 2 mM l-glutamine, 20 mM HEPES (pH 7.5), streptomycin, and penicillin.

### Sequence analysis and identification of the TgCaMKrk open reading frame (ORF)

*T. gondii* RH strain mRNA was isolated from infected Vero cells by using TRIZOL (Invitrogen, Carlsbad, CA, USA) according to the manufacturer’s instructions. A cDNA library of *T. gondii* RH strain was amplified by RT-PCR using parasite mRNA as the template and SuperScrip III Reverse Transcriptase with oligo dT primer (Invitrogen, Carlsbad, CA, USA). The TgCaMKrk ORF was identified by sequence analysis of the *T. gondii* cDNA library by using an Applied Biosystems 3130 Genetic Analyzer.

### Plasmids

**Fig. 1:**
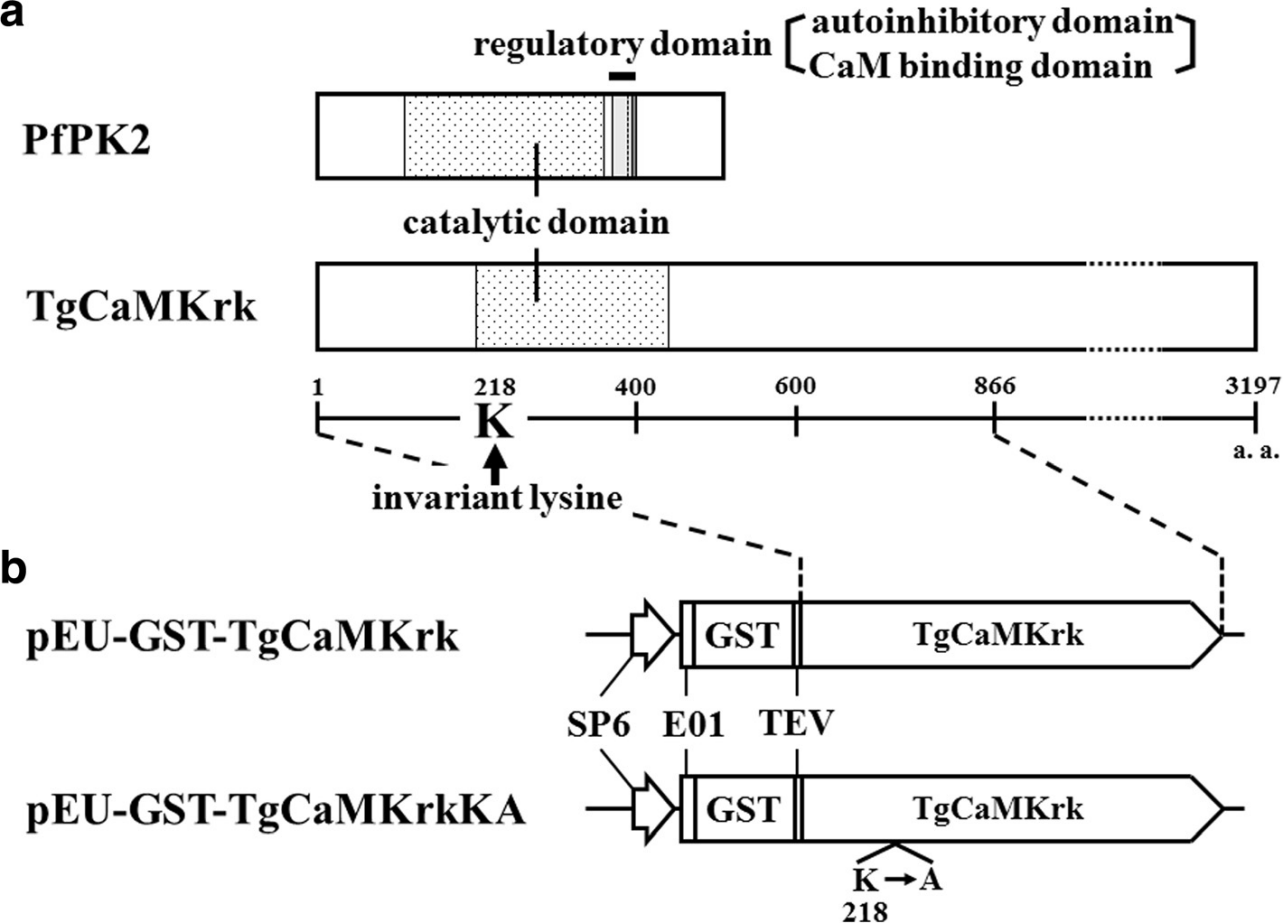
Diagrams of the putative amino acid sequences of PfPK2 and TgCaMKrk, and of the expression plasmids used in this study. **a** The amino acid sequences of PfPK2 and TgCaMKrk are shown; the catalytic and regulatory domains, including the autoinhibitory and calmodulin-binding domains [27], are shaded. TgCaMKrk has no regulatory domain. The arrow denotes the conserved catalytic lysine. **b** The plasmids (pEU-GST-TgCaMKrk and pEU-GST-TgCaMKrkKA) used for the expression of GST-TgCaMKrk and GST-TgCaMKrkKA, respectively, in the wheat germ cell-free protein synthesis system are shown. The SP6 promoter (SP6), translational enhancer (E01), GST, and tobacco etch virus protease recognition site are indicated [36]

The TgCaMKrk gene encoding the predicted kinase domain (amino acids [a.a.] 1–866) was amplified by RT-PCR using parasite mRNA as the template and the following primers: forward, 5'-GCG CCT CGA GGG AGA AGT TTT TGG CGC TTT-3' (*Xho*I digestion site is underlined), and reverse, 5'-GCA CTA GTC TAC ACG TGA CGA AGT GGA A-3' (*Spe*I digestion site is underlined). The product was digested with *Xho*I/*Spe*I and cloned into pEU (CellFree Sciences, Yokohama, Japan) to generate a GST fusion protein. The resultant plasmid was designated pEU-GST-TgCaMKrk (Fig. 1b). To generate pEU-GST-TgCaMKrkKA, Lys^218^ of TgCaMKrk was replaced with Ala by using a QuikChange Site-Directed Mutagenesis Kit (Stratagene, Santa Clara, CA, USA) with the oligonucleotide 5'-GAG AAG GTC GTT GTC GCG GCC ATC AAC AAA AAG-3' and its complement, according to the manufacturer’s instructions. The complete ORFs of TgGAP45, TgACT1, TgMLC1, and TgGAP50 were amplified from parasite mRNA by RT-PCR using the primers listed in Table 1. The amplified fragments were digested with the restriction enzymes indicated in Table 1 and cloned in-frame into pMal-c (New England BioLabs, Beverly, MA, USA) with maltose-binding protein (MBP) to generate pMal-TgGAP45, pMal-TgACT1, pMal-TgMLC1, and pMal-TgGAP50, respectively. Simultaneously, a TgGAP45 mutant plasmid that lacked most of the coiled-coil domain (a.a. 27–136) [24], was also generated (refer to Fig. 6a).

**Table 1:** The list of primers used to generate the expression vectors

Expression plasmid	Direction	Sequence
pMal-TgGAP45	Forward ^a^	5′-GCGAATTCGGAAACGCGTGCAAGAAGAA-3′
	Reverse ^b^	5′-GCCTGCAGTCAGTTCAACAAGGGTGCAT-3′
pMal-TgACT1	Forward ^a^	5′-GCGAATTCGCGGATGAAGAAGTGCAAGC-3′
	Reverse ^c^	5′-GCTCTAGATTAGAAGCACTTGCGGTGGA-3′
pMal-TgMLC1	Forward ^a^	5′-GGAATTCGCGAGCAAGACCACGTCTG-3′
	Reverse ^c^	5′-GCTCTAGACTAGAACGCCGGCTGAACAG-3′
pMal-TgGAP50	Forward ^d^	5′-GGGGTACCGGCAGGCGCCCCCGTCGCG-3′
	Reverse ^c^	5′-GCTCTAGATTATTTCATGTAGCGAGAG-3′

^a^ *Eco*RI site is underlined. ^b^ *Pst*I site is underlined. ^c^ *Xba*I site is underlined. ^d^ *Kpn*I site is underlined

### Wheat germ cell-free protein synthesis system

Protein expression using a wheat germ cell-free protein synthesis system (CellFree Sciences) was achieved as described previously [27, 28]. Briefly, at the transcription step, 2 μg of pEU-GST-GFP or pEU-GST-TgCaMKrk was mixed with 18 μl of transcription mixture (transcription buffer with 2.5 mM NTP mix, 1 U/μl RNase inhibitor, and 1 U/μl SP6 RNA polymerase; CellFree Sciences) and incubated at 37 °C for 6 h. Each mRNA generated was then mixed with 10.8 μl of WEPRO1240G (CellFree Sciences) and 40 ng/μl creatine kinase (Roche, Mannheim, Germany), transferred to the bottom of the SUB-AMIX solution (CellFree Sciences) to form a bilayer, and incubated at 16 °C for 20 h. To determine whether the resultant protein was bound to Ca^2+^ or calmodulin upon translation, DMSO (negative control), the calmodulin antagonist W-7 (0.66 mM; BIOMAL, Plymouth Meeting, PA, USA), or calmidazolium (0.05 mM; Sigma-Aldrich, St. Louis, MO, USA) was added to the SUB-AMIX solution.

### Purification of recombinant proteins

Wheat germ extracts were mixed with 10 μl of a 50 % slurry of glutathione-Sepharose beads (GE Healthcare, Little Chalfont, Buckinghamshire, UK) for 16 h. The beads were then washed three times with phosphate-buffered saline (PBS). Purified protein captured on the beads was separated by 10 % sodium dodecyl sulfate-polyacrylamide gel electrophoresis (SDS-PAGE) after boiling and was then either silver stained or immunoblotted with anti-α-GST antibodies (Sigma-Aldrich). Purification of the recombinant proteins expressed in *Escherichia coli* XL-1 blue transformed with plasmids harboring MBP-TgGAP45, MBP-TgACT1, MBP-TgMLC1, or MBP-TgGAP50 was performed as described elsewhere [29].

### In vitro kinase assay

Purified GST-GFP, GST-TgCaMKrk, or GST-TgCaMKrkKA captured on glutathione-Sepharose beads was rinsed twice with washing buffer (50 mM Tris-HCl, pH 9.0, and 2 mM DTT). Kinase assay reactions were performed with the purified GST fusion proteins at 37 °C for 30 min in 50 μl of kinase buffer (50 mM Tris-HCl, pH 8.0, 200 mM NaCl, 50 mM MgCl_2_, 0.1 % Nonidet P-40, 1 mM DTT, 5 μM ATP, and 10 μM CaCl_2_) containing 5 μCi of [γ-^32^P]ATP. After incubation, the samples were washed three times with TNE buffer (20 mM Tris-HCl, pH 8.0, 100 mM NaCl, and 1 mM EDTA), and the phosphorylated proteins were separated by 10 % SDS-PAGE. The gels were stained with Coomassie Brilliant Blue (CBB), dried, and exposed to X-ray film [27, 28].

### Phosphatase treatment

After in vitro kinase assays, GST fusion proteins captured on glutathione-Sepharose beads were subjected to phosphatase treatment, as described elsewhere [27, 28].

### Antibodies

Rabbit anti-GRA6 polyclonal antibodies were kindly sent to us by Dr. L.D. Sibley (Washington University School of Medicine, St. Louis, MO, USA); rabbit anti-M2AP (microneme protein 2-associated protein) polyclonal antibodies were kindly provided by Dr. V. Carruthers (John Hopkins University, Baltimore, MD, USA); rabbit anti-ROP1 polyclonal antibodies were a gift from Dr. J. Dubremetz (University of Montpellier, Montpellier, France); and rabbit anti-TgGAP45 polyclonal antibodies were a gift from Dr. D. Soldati (University of Geneva, Switzerland).

### Production of anti-TgCaMKrk antibodies

Mouse antiserum against TgCaMKrk was prepared by immunizing BALB/c mice with purified GST-TgCaMKrk expressed in a wheat germ cell-free system, purified on glutathione-Sepharose beads, extensively washed with buffer C, and eluted with elution buffer (10 mM glutathione and 500 mM Tris-HCl, pH 8.0). For the first immunization, a mixture of the eluted supernatants and Freund’s complete adjuvant (Rockland, Gilbertsville, PA, USA) was injected intraperitoneally into the mice. After 2 weeks, a mixture of the eluted supernatants and Freund’s incomplete adjuvant (Rockland) was injected three times, at 2-week intervals. One week after the final injection, mouse antiserum against TgCaMKrk was collected.

### Immunoblotting and IFA

*T. gondii* RH strain was propagated and lysed by passing the cells though a #27 syringe and was filtered by using a 5-μm filter. The lysate was subjected to immunoblotting as described elsewhere [30]. For indirect immunofluorescence microscopy (IFA), purified extracellular tachyzoites were fixed on a 14-well slide with 40 % formaldehyde and were permeabilized with 0.1 % Triton X-100. The slide was incubated with antibodies against TgCaMKrk and GRA6, TgGAP45, ROP-1, or M2AP, respectively, for 60 min at 37 °C and then rinsed three times with PBS. After incubation with AlexaFluor 488-conjugated goat anti-mouse IgG (H + L) and AlexaFluor 546-conjugated goat anti-rabbit IgG (H + L) (Invitrogen) for 60 min at room temperature, the slide was rinsed three times with PBS. Intracellular tachyzoites were prepared by infecting Vero cells cultured in an eight-well chamber slide for 48 h; the slide was then fixed and stained as described above.

## Results

### Identification of TgCaMKrk

We previously characterized PfPK2, a unique CaMK encoded by the *P. falciparum* genome [27]. Calmodulin antagonists inhibited PfPK2 kinase activity in vitro and markedly decreased ring-stage parasitemia in invasion assays [27]. CaMK plays important roles in Ca^2+^ signaling during various cellular events, including carbohydrate metabolism, transcription, spermatogenesis, transcription, neuronal memory, and mitochondrial biogenesis [31]. Therefore, we speculated that such a gene likely exists in another apicomplexan parasite, *T. gondii*. In this study, we sequenced and identified an ORF (9,591 bp) of a novel *T. gondii* gene that included a sequence homologous to the catalytic domain of PfPK2. These sequence analysis data were consistent with transcriptional data from the RNA-seq of *T. gondii* ME49 strain performed by Drs. J. Yamagishi and X. Xuan (Obihiro University of Agriculture and Veterinary Medicine; personal communication). The putative catalytic domain of TgCaMKrk shares 57 % identity and 88 % similarity with that of PfPK2; however, it has no homologous regulatory (autoinhibitory and calmodulin-binding) domain and a long nonconserved region (Fig. 1a). We named this gene *T. gondii* CaMK-related kinase (TgCaMKrk) (GenBank accession number: AB699221).

### Purification of the TgCaMKrk kinase domain by using a wheat germ cell-free protein synthesis system

**Fig. 2:**
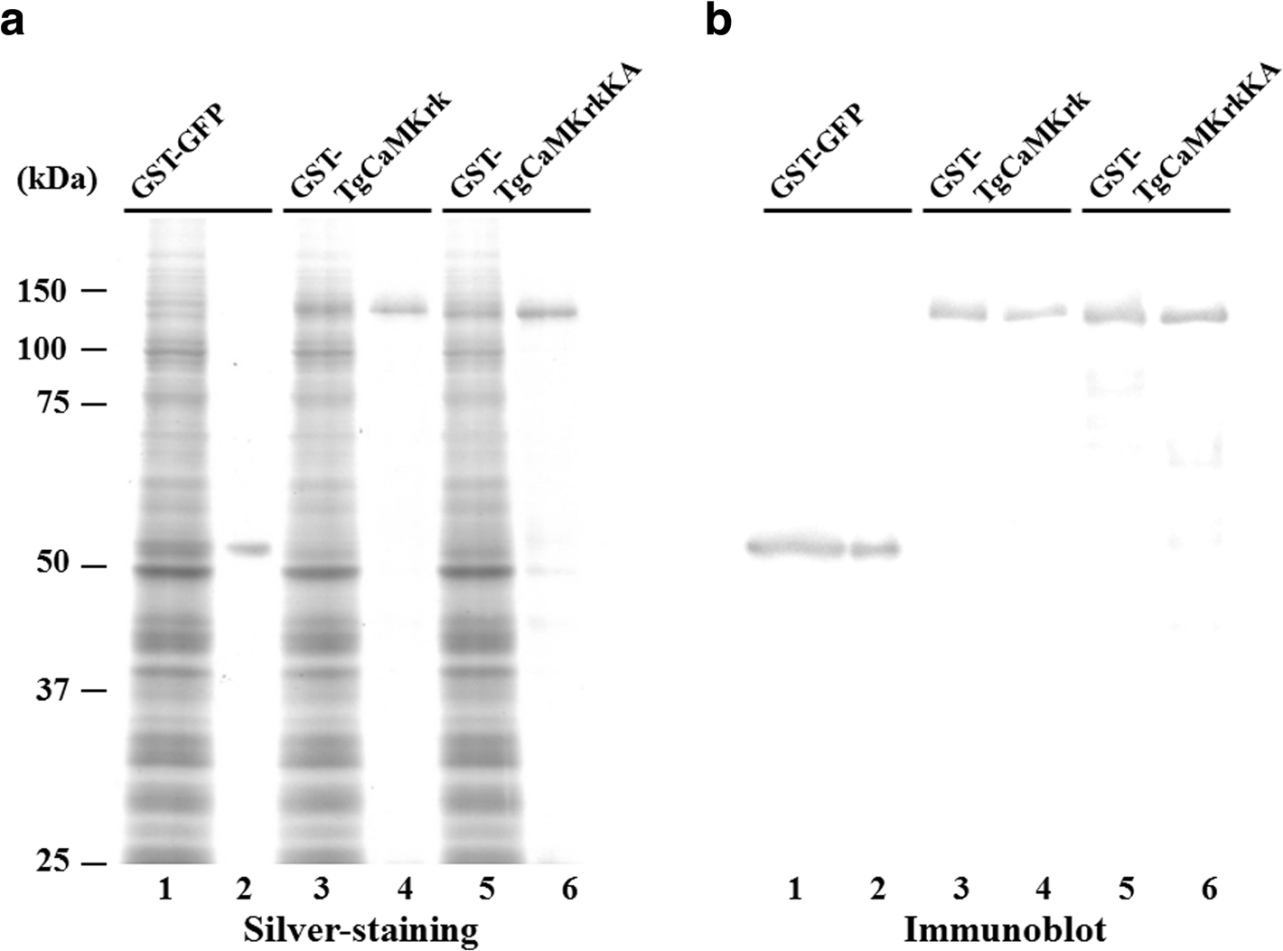
Expression and purification of GST-GFP, GST-TgCaMKrk, and GST-TgCaMKrkKA. Proteins were expressed by using a wheat germ cell-free protein synthesis system. Total wheat germ extracts were subjected to affinity chromatography using glutathione-Sepharose beads. The purified proteins were separated on denaturing gels and then (**a**) silver stained or (**b**) transferred to a nitrocellulose sheet and probed with anti-GST antibodies. Lanes 1 and 2, GST-GFP; Lanes 3 and 4, GST-TgCaMKrk; Lanes 5 and 6, GST-TgCaMKrkKA. Lanes 1, 3, and 5, total wheat germ extracts; Lanes 2, 4, and 6, purified proteins. Molecular masses (kDa) are shown on the left

Our first objective was to purify the *TgCaMKrk* gene product. However, the putative protein (3,197 amino acids) was too large to be expressed in vitro. Therefore, we expressed and purified the TgCaMKrk kinase domain (N-terminal 866 amino acids), which includes the catalytic domain, as a glutathione S-transferase (GST) fusion protein by using a wheat germ cell-free protein synthesis system (Fig. 1b). The kinase domain was selected because no other conserved domains were found during our BLAST analysis of the TgCaMKrk putative amino acids. The purified protein was then separated by denaturing gel electrophoresis and either silver stained (Fig. 2a) or immunoblotted with anti-GST antibodies (Fig. 2b). The purified protein products, GST-GFP, GST-TgCaMKrk, and GST-TgCaMKrkKA, each contained one major band (*M*_r_ values of 54,000, 121,000 and 121,000, respectively) and reacted with antiserum containing anti-GST antibodies (Fig. 2b).

### Protein kinase activity of TgCaMKrk

**Fig. 3:**
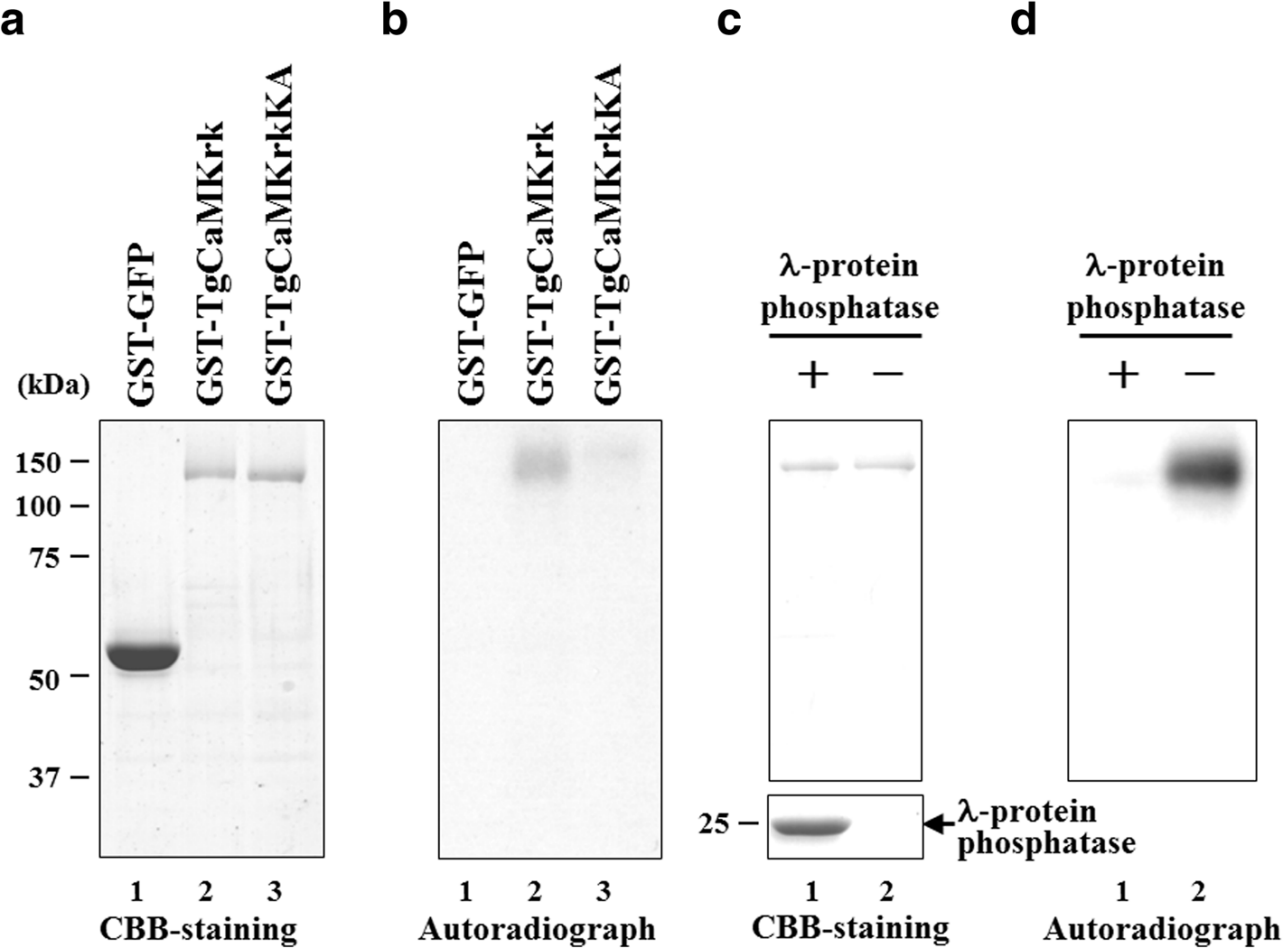
In vitro kinase assays of purified GST-GFP, GST-TgCaMKrk, and GST-TgCaMKrkKA. **a** Purified GST-GFP (Lane 1), GST-TgCaMKrk (Lane 2), or GST-TgCaMKrkKA (Lane 3) was incubated in kinase buffer containing [γ-^32^P]ATP, separated on a denaturing gel, and stained with CBB. **b** Autoradiograph of the gel shown in **a**. **c** Purified GST-TgCaMKrk incubated in kinase buffer containing [γ-^32^P]ATP (Lane 1) and the labeled protein treated with λ-protein phosphatase (Lane 2) were separated on a denaturing gel and then stained with CBB. **d** Autoradiograph of the gel shown in **c**. Molecular masses (kDa) are indicated on the left

Many protein kinases exhibit autophosphorylation activity [32]. To determine whether TgCaMKrk has such activity, we tested the purified protein in a kinase assay. Purified fusion proteins were incubated in kinase buffer containing [γ-^32^P]ATP, separated electrophoretically, and stained with CBB (Fig. 3a and b). Purified GST-GFP fusion protein, which was incubated in kinase buffer and electrophoretically separated, did not contain any labeled bands (Fig. 3a and b, Lanes 1). However, in the autoradiographic image of purified TgCaMKrk, a protein band with an apparent *M*_r_ of 121000 was labeled (Fig. 3b, Lane2). The electrophoretic mobility of labeled TgCaMKrk was the same as that of purified TgCaMKrk stained with CBB (Fig. 3a and b). To confirm that the [γ-^32^P]ATP labeling of TgCaMKrk was due to autophosphorylation, labeled TgCaMKrk was boiled (to inactivate its kinase activity) and incubated with 5 U of alkaline phosphatase at 37 °C for 30 min. No band corresponding to labeled TgCaMKrk was detected after this phosphatase treatment, indicating that TgCaMKrk was indeed labeled with [γ-^32^P]ATP by phosphorylation (Fig. 3c and d). Importantly, the total TgCaMKrk protein levels were similar before and after boiling. Thus, TgCaMKrk possesses protein kinase activity and is able to phosphorylate itself.

It is possible that the kinase activity we detected in these experiments was caused by a contaminating kinase(s) that physically associates with GST-TgCaMKrk, or that it was non-specifically selected for by the glutathione-Sepharose beads. To exclude these possibilities, we constructed a mutant that lacked intrinsic kinase activity but retained its overall structure. We used site-directed mutagenesis to replace Lys^218^ of TgCaMKrk with an Ala residue to generate the GST fusion protein GST-TgCaMKrkKA (see Fig. 1b). Lys^218^ was chosen because it is conserved in subdomain II of known protein kinases and is required for kinase activity [33], and because mutation of the corresponding lysine in PfPK2 resulted in a loss of kinase activity [27]. The autophosphorylation activity of GST-TgCaMKrkKA was tested as before using purified protein and [γ-^32^P]ATP. The wild-type fusion protein was labeled by autophosphorylation (Fig. 3b, Lane 2), whereas the mutant was not (Fig. 3b, Lane 3). These results indicate that the substitution of Lys^218^ abolished the kinase activity of TgCaMKrk and that contaminating kinases were not a factor in our kinase assay.

### Neither Ca^2+^ nor calmodulin are required for the protein kinase activity of TgCaMKrk

**Fig. 4:**
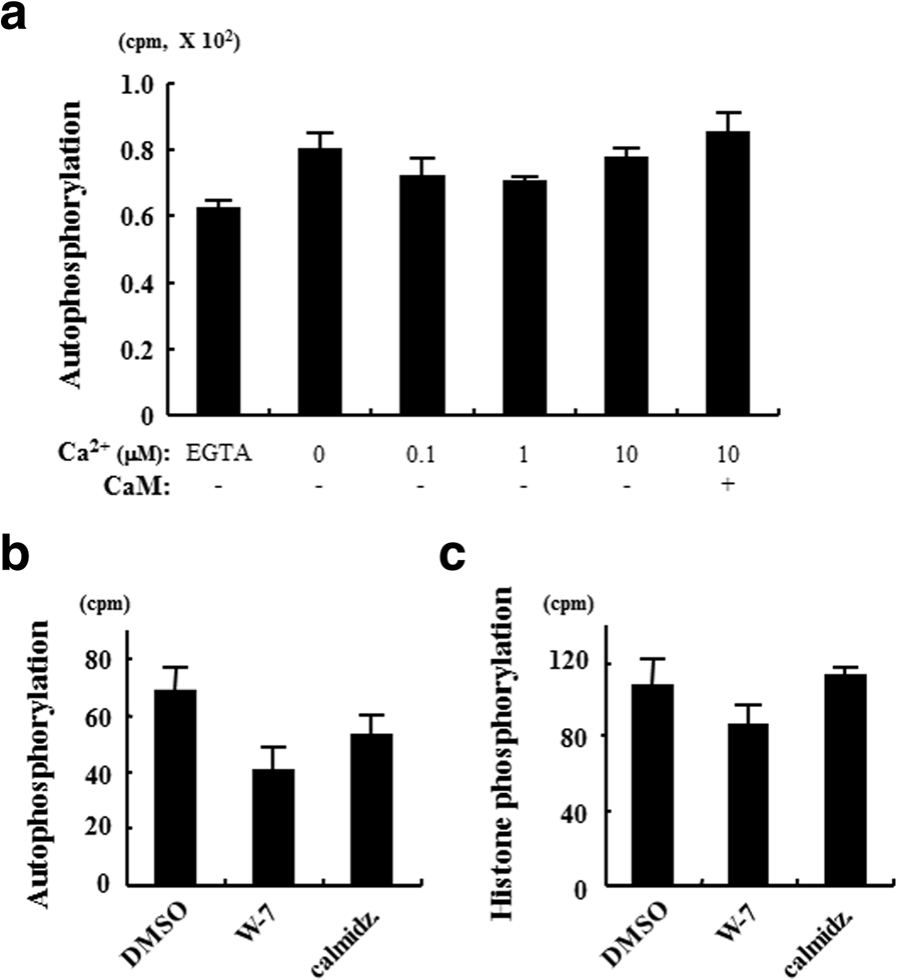
Characterization of the kinase activity of TgCaMKrk. **a** In vitro kinase assays were performed using purified GST-TgCaMKrk and histone II_AS_ in the presence of EGTA or 0–10 μM Ca^2+^, or 10 μM calmodulin and 10 μM Ca^2+^. The autophosphorylation of TgCaMKrk was measured by scintillation counting. Effect of calmodulin antagonists on the (**b**) autophosphorylation and (**c**) histone II_AS_ phosphorylation activity of TgCaMKrk in the presence of DMSO or the calmodulin antagonists W-7 and calmidazolium (calmidz.) in a wheat germ cell-free system. The autophosphorylation and histone phosphorylation activity of TgCaMKrk were measured by scintillation counting. Representative data from three independent experiments are shown

To determine whether the kinase activity of TgCaMKrk is Ca^2+^ and/or calmodulin dependent, as was shown for PfPK2 [27], we performed two series of experiments (Fig. 4a). First, in vitro kinase assays were performed in the presence of EGTA or 0–10 μM Ca^2+^. The kinase activity of TgCaMKrk was the same, regardless of the presence of Ca^2+^. Next, we assessed the effect of Ca^2+^ and calmodulin on the phosphorylation activity of TgCaMKrk. We found that TgCaMKrk activity was not affected by the presence of 10 μM Ca^2+^ or 10 μM calmodulin, while the autophosphorylation of PfPK2 depended on the calcium concentration.

We also tested the effect of calmodulin antagonists on the phosphorylation activity of TgCaMKrk, because we had previously demonstrated their inhibition of PfPK2 activity in vitro and of erythrocyte invasion by *P. falciparum* [27]. The translation mixture (i.e. WEPRO1240G) used in the wheat germ cell-free system contained wheat calmodulin, so we eliminated the wheat calmodulin at the translation step by incubating the translation mixture with the calmodulin antagonist W-7 or calmidazolium. After translation and purification, TgCaMKrk activity was assessed by scintillation counting. Both autophosphorylation and histone phosphorylation were slightly reduced after the addition of W-7 (Fig. 4b and c), while the activity of autophosphorylation of PfPK2 was greatly reduced after the addition of W-7. Thus, neither Ca^2+^ nor calmodulin is required for the kinase activity of TgCaMKrk. These results are consistent with the fact that the putative amino acid sequence of TgCaMKrk has a conserved catalytic domain but lacks the regulatory domain, which comprises the autoinhibitory and calmodulin-binding domains in CaMK-like proteins (Fig. 1a) [33].

### TgCaMKrk is expressed at the periphery of *T. gondii* tachyzoites

**Fig. 5:**
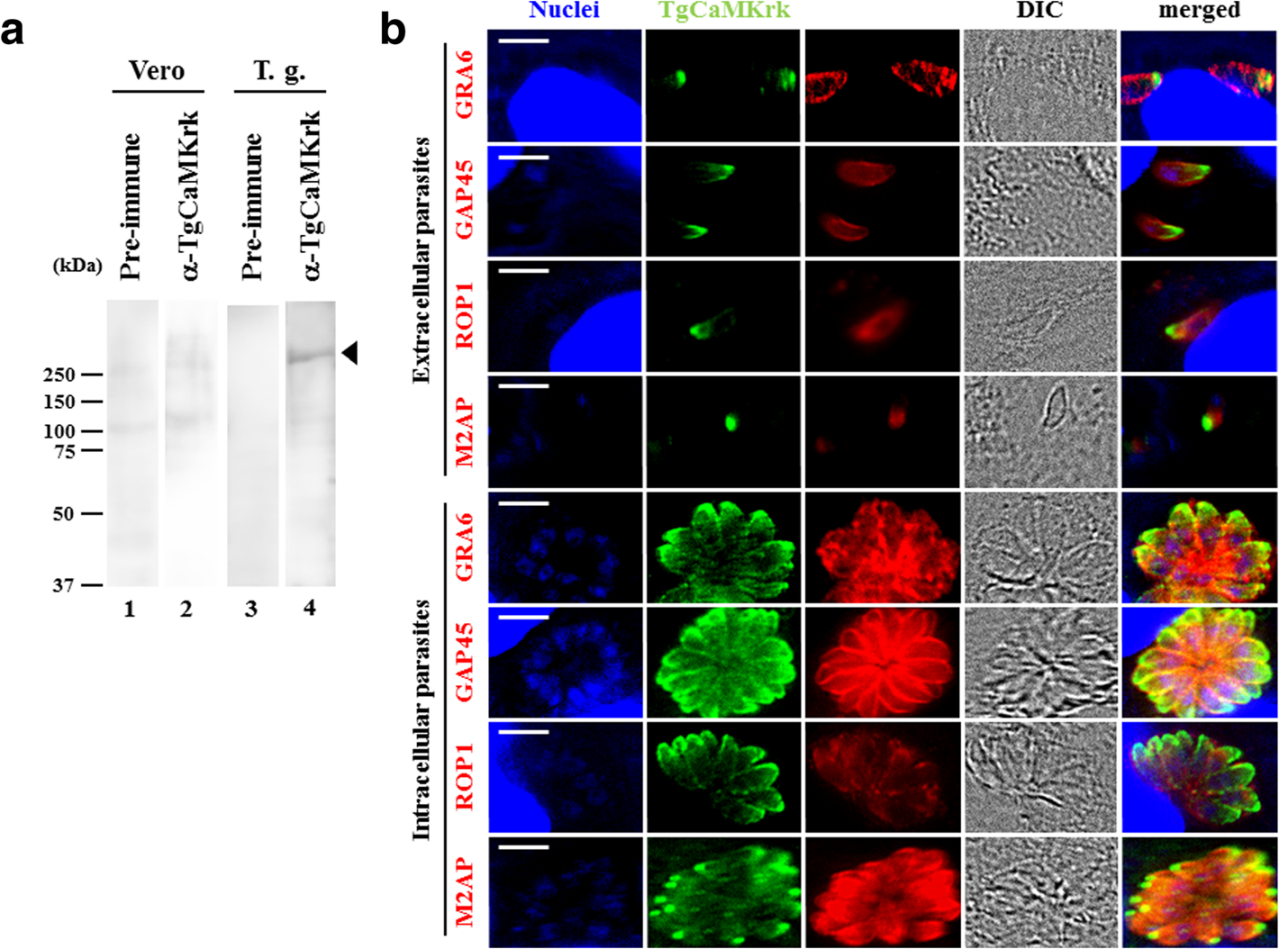
Expression of TgCaMKrk in *T. gondii*-infected cells. **a** Immunoblotting was performed using lysates from *T. gondii* (Lanes 1 and 2) or Vero cells (Lanes 3 and 4) and anti-TgCaMKrk mouse serum (Lanes 2 and 4) or pre-immune mouse serum (Lanes 1 and 3). The arrowhead indicates expression of TgCaMKrk. Molecular masses (kDa) are indicated on the left. **b** Localization of TgCaMKrk in *T. gondii*. Mouse anti-TgCaMKrk antibody was used as the primary antibody, and anti-GRA6, -GAP45, -ROP1 and -M2AP antibodies from rabbits were used as markers of parasite organelles. Nuclei are stained with TO-PRO3. The four upper panels indicate the extracellular parasite; the four lower panels indicate the intracellular *T. gondii*. Differential interference contrast (DIC) images and overlaid images are shown in the two right panels. *Scale-bar*: 5 μm

To investigate the localization of TgCaMKrk, lysates of *T. gondii* tachyzoites were immunoblotted with antibodies raised against GST-TgCaMKrk in mice (Fig. 5). A single band was detected at approximately 340 kDa, which corresponds to the predicted molecular weight of TgCaMKrk (Fig. 5a, Lane 4). No band was detected in an uninfected Vero cell lysate (Fig. 5a, Lanes 1 and 2).

PfPK2 is expressed in the membrane of the parasite [34]. Our previous IFA data showed that PfPK2 is produced by merozoites during erythrocyte invasion [27]. To determine the localization of the TgCaMKrk protein in *T. gondii*, rabbit anti-GRA6, -GAP45, -ROP1 and -M2AP antibodies were employed to show the dense granules, glideosome, rhoptry, and apical ends. As shown in Fig 5b, in the released tachyzoite, the TgCaMKrk signal did not completely co-localize with any of the markers we used, but it was located more toward the apical end of the M2AP signal and closest to the GAP45 signal (Fig. 5b, 2nd panel). In the growing tachyzoites in the infected cells, the TgCaMKrk signal localized to the apical ends and again did not completely co-localize with any of the marker signals (Fig. 5b, 5th–8th panels). Pre-immune mouse serum did not stain any parasites (data not shown). The TgCaMKrk signal at the more apical end of M2AP suggests an apical end location for TgCaMKrk; however, further study is required to determine the precise localization of this protein.

### TgCaMKrk specifically phosphorylates the glideosome protein TgGAP45 in vitro

**Fig. 6:**
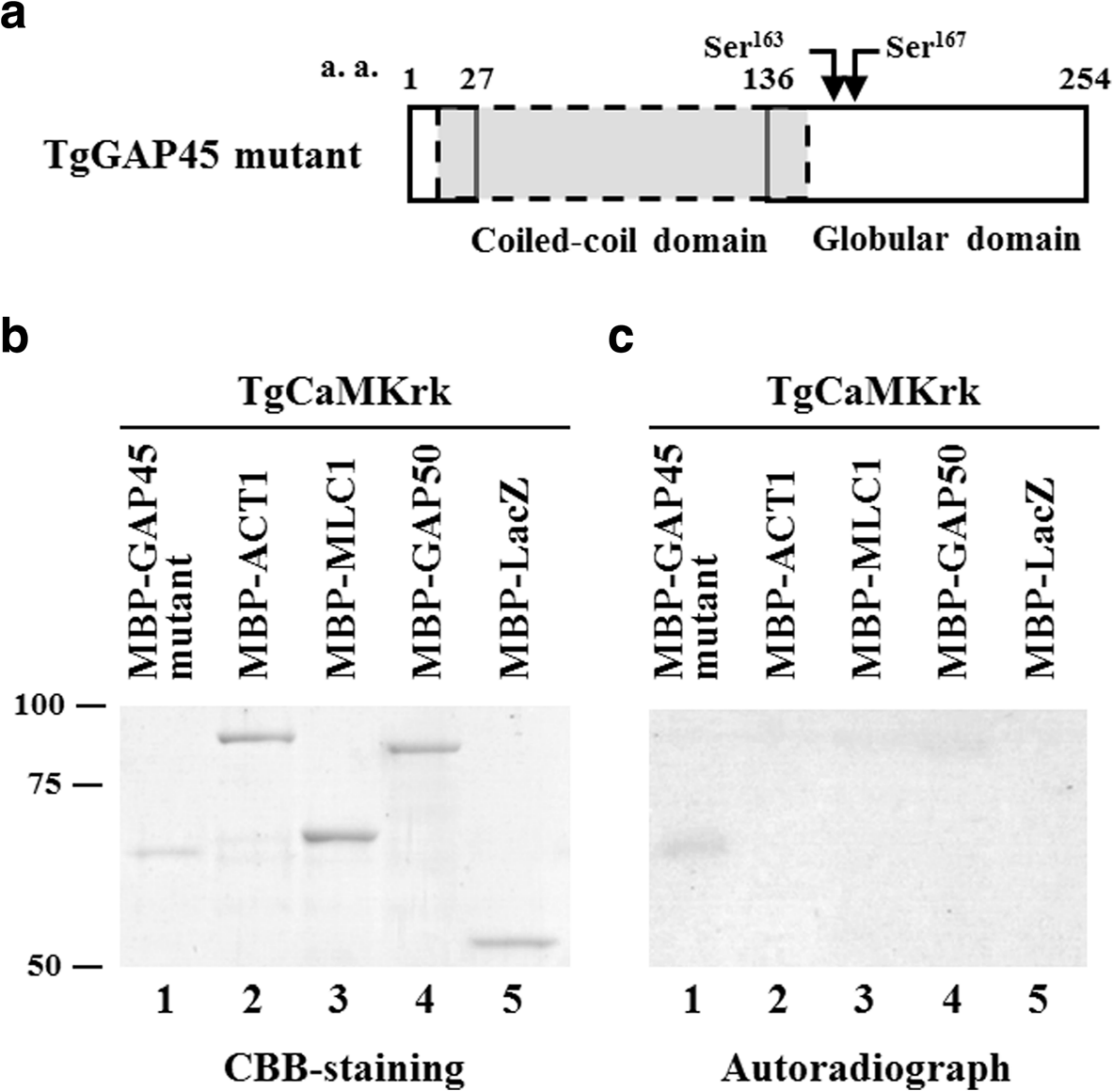
In vitro kinase assay of *T. gondii* component proteins with TgCaMKrk. **a** A schematic diagram of the TgGAP45 domain structure and its deletion mutant is shown. TgGAP45 consists of an N-terminal coiled-coil domain (*shaded*) and a C-terminal globular domain. The two phosphorylation sites, Ser^163^ and Ser^167^ [24], are indicated by arrows. The TgGAP45 mutant has a deletion in its coiled-coil domain (a.a. 27–136). **b** MBP-GAP45 mutant (predicted MW, 59 kDa; Lane 1), MBP-ACT1 (84 kDa; Lane 2), MBP-MLC1 (66 kDa; Lane 3), MBP-GAP50 (88 kDa; Lane 4), and MBP-LacZ (51 kDa; Lane 5) were immobilized on amylose resin beads and subjected to in vitro kinase assays with TgCaMKrk. The proteins were then washed three times with TNE buffer, separated on a denaturing gel, and stained with CBB. **c** An autoradiograph of the gel shown in A. Molecular masses (kDa) are indicated on the left

*T. gondii* and *P. falciparum* protein kinases phosphorylate glideosome component proteins, including GAP45 and MTIP [24, 26]. Because mass spectroscopy identified residues Ser^163^ and Ser^167^ of TgGAP45 as phosphorylated residues [24], we used a deletion mutant of TgGAP45 that contain the globular domain with the predicted kinase-target residues Ser^163^ and Ser^167^ (Fig. 6a). As we obtained the data that TgCaMKrk signal is localized at the apical end, the interaction between TgCaMKrk and glideosome components should be analyzed. We selected glideosome components, TgACT1, TgMLC1, and TgGAP50 including TgGAP45 [20] as subjects of interest. To determine which glideosome component TgCaMKrk phosphorylates, we performed in vitro kinase assays using a TgGAP45 deletion mutant, TgACT1, TgMLC1, TgGAP50, and LacZ as substrates (Fig. 6b and c). Of the tested proteins, only TgGAP45 was phosphorylated by TgCaMKrk in vitro.

## Discussion

This is the first report to describe the identification and characterization of a CaMK-like protein in *T. gondii*, TgCaMKrk. GST-TgCaMKrk containing the kinase domain was successfully expressed and purified by using a wheat germ cell-free protein synthesis system. TgCaMKrk was shown to have both autophosphorylation and substrate phosphorylation activity, as well as a conserved catalytic lysine (Lys^218^). The catalytic domain of TgCaMKrk shares 88 % similarity with that of PfPK2; however, TgCaMKrk has no regulatory domain. The regulatory domain of PfPK2 consists of autoinhibitory and calmodulin-binding domains. The lack of such a domain in TgCaMKrk is reflected by the fact that calmodulin antagonists (W-7 and calmidazolium) did not inhibit TgCaMKrk autophosphorylation or the histone phosphorylation activity of TgCaMKrk. Some regulation of TgCaMKrk must occur, however, because continuous phosphorylation by this protein would presumably affect the parasite’s life-cycle. A BLAST search via ToxoDB (http://toxodb.org/toxo/) using the putative amino acid sequence of TgCaMKrk did not reveal a domain homologous to the regulatory domain of PfPK2. Therefore, it is likely that another parasite protein regulates the kinase function of TgCaMKrk.

Our IFA data showed that TgCaMKrk localizes to the apical ends of extracellular tachyzoites. The localization patterns of TgCaMKrk in extracellular tachyzoites were more posterior to those of GAP45. Antibodies against TgGAP45 stained the inner membrane complex, but not the plasma membrane [35]. Previous fractionation analysis of *P. falciparum* indicated that PfPK2 is also expressed in the parasite’s inner membrane [34]. These findings suggest that TgCaMKrk could be secreted as it is associated with apical organelles, and it could have activity against host proteins for pathogenesis, and localize with a specific organelle in the inner membrane complex in tachyzoites.

TgCaMKrk specifically phosphorylated TgGAP45 in the glideosome in vitro. The residues of TgGAP45 phosphorylated by TgCaMKrk were most likely Ser^163^ and Ser^167^. Mutation of these residues to Glu prevented association of the MyoA-MLC1-GAP45 complex with GAP50, but did not have any obvious effects on motility or host cell invasion, suggesting that Ser^163^ and Ser^167^ phosphorylation controls the final step in assembly of the myosin XIV motor complex [24].

TgGAP70 localized to the apical cap of extracellular tachyzoites, whereas TgGAP45 localized to the periphery [22]. When we examined the homology between TgGAP45 and TgGAP70, we found that the similarity was approximately 73 % (Genetyx). However, the critical serine residues corresponding to Ser^163^ and Ser^167^ of TgGAP45 are not conserved in TgGAP70. Further analysis of TgCaMKrk in extracellular tachyzoites is needed to clarify its substrates.

*T. gondii* kinases play important and diverse roles in the parasite’s life-cycle [15]. Inhibitor studies suggest that kinases are crucial regulators of parasite invasion. Therefore, such proteins represent good potential drug targets for blocking parasite-specific activity. On the basis of the data presented here, we suggest that TgCaMKrk should be explored as a drug target, because it localizes to the apical ends of *T. gondii* tachyzoites and could be associated with the glideosome. Future studies should focus on the regulatory mechanism for this protein kinase and its overall impact on the life-cycle of *T. gondii*.

## Conclusion

In this study, we identified TgCaMKrk, which exhibits autophosphorylation and histone phosphorylation activity. TgCaMKrk is expressed in *T. gondii*-infected cells and localizes to the apical ends of extracellular and intracellular tachyzoites. TgCaMKrk phosphorylates TgGAP45 in vitro. Our data further our understanding of *T. gondii* motility and infection, the interaction between parasite protein kinases and glideosomes, and drug targets for protozoan diseases.
